# Soil legislation and policies: Bibliometric analysis, systematic review and quantitative approaches with an emphasis on the specific cases of the European Union and Portugal

**DOI:** 10.1016/j.heliyon.2024.e34307

**Published:** 2024-07-09

**Authors:** Vítor João Pereira Domingues Martinho, António José Dinis Ferreira, Carlos Cunha, José Luís da Silva Pereira, María del Carmen Sánchez-Carreira, Nádia Luísa Castanheira, Tiago Brito Ramos

**Affiliations:** a School of Agriculture (ESAV) and CERNAS-IPV Research Centre, Polytechnic Institute of Viseu, Portugal; b School of Agriculture (ESAC) and CERNAS Research Centre, Polytechnic Institute of Coimbra, Portugal; c School of Technology and Management (ESTGV), Polytechnic Institute of Viseu, Portugal; d ICEDE Research Group, Applied Economics Department, Faculty of Economics and Business Sciences, CRETUS, Universidade de Santiago de Compostela, Spain; e National Institute of Agricultural and Veterinary Research, IP (INIAV), Portugal; f MARETEC, Instituto Superior Técnico, Universidade de Lisboa, Portugal

**Keywords:** Bibliographic data, Qualitative data, Soil strategy, Word cloud

## Abstract

The literature shows that there are dimensions related to soil legislation and policy in the European Union contexts that can be better explored through bibliometric analysis, systematic reviews and quantitative approaches. Therefore, this research aims to analyse documents on soil legislation and policies, highlighting the specific cases of Portugal and the European Union (EU). The aim is to identify suggestions to improve the Portuguese and European Union soil policy instruments and measures. To achieve these objectives, a bibliometric analysis (considering text and bibliographic data) and systematic review were carried out, as well as a survey of the available soil legislation (considering qualitative data and quantitative analysis). The results show that soil legislation and policy have become more relevant in recent years and that concerns are about soil health, protection and safety, as well as risk mitigation, biodiversity preservation and the maintenance of ecosystem services. However, some topics could be further explored in future research, namely those related to multidisciplinarity, smart methodologies, soil salinisation, innovation and quantitative approaches to assessing policy impacts. This study presents suggestions that can be considered by the Portuguese and European Union policymakers to improve the respective soil legislation and policies. Defining a regulatory system for soils in the European Union has not been easy over time, although there have been attempts, given the specificities of the contexts related to soils and the reluctance of some member states to take certain measures. The approaches and analysis topics considered are innovative (there aren't many scientific documents on the topics that address bibliometric analysis and quantitative assessments with qualitative data) and bring novelty to the literature.

## 1 Introduction

To pursue sustainable development, it is required to establish more compatible and balanced interrelationship between economic, social and environmental dimensions. It is also crucial to establish a continuous assessment of the availability and quality of strategic resources, such as the soil, where the conditions of use have major implications [1]. In these contexts, legislation and policies are fundamental to protect the soil quality and mitigate risks and threats as a consequence of socioeconomic activities. For that, the European Union (EU) may need to rethink its soil legal framework to make the legislation more effective and adjusted to the current challenges. Policy initiatives outlined in the European Green Deal may also be strengthened to better protect soils [2].

In any case, the new proposal for a Directive on Soil Monitoring and Resilience, the EU Soil Strategy 2030, and the EU Soil Observatory may bring important contributions to promote more sustainable soil management [3]. These EU policies and strategies have their impacts on the different member-states′ contexts [4]. On the other hand, concerns about environmental quality increased over the last decades [5]. Soil threats are, in general, linked with compaction, salinisation, sealing, erosion, level of organic matter, decline in biodiversity and contamination. In some circumstances, EU soil policies face challenges in comprehensively addressing current threats [6]. In these frameworks, the EU aims to protect the following soil functions [7]: biomass supply; water quality improvement; biodiversity preservation; raw materials supply; construction support; carbon sequestration; and cultural heritage preservation. Nonetheless, to achieve these aims, the EU needs to deal with the soil dimensions from a wider perspective. It would also be interesting to consider the soil-water system, in addition to concerns about soil protection and remediation [8]. These approaches may be relevant to better understand the dynamics and costs of soil erosion. Soil erosion is one of the main factors with implications on land conditions [9]. Data and information are crucial in the designing process of adjusted soil policies [10,11], but, in some cases, a standardisation of methods and concepts is needed [12]. Equally important is a deeper understanding of the ways these policies interact [13]. Adjustments in the Common Agricultural Policy framework, for instance, may contribute to more sustainable land management [14]. The design of common EU policies and strategies is not always easy [15], due to particularities of each member-state [16].

In the Portuguese context, in terms of legislation and policy, air and water quality were prioritised to the detriment of the soil protection and remediation. This scenario has created serious difficulties for sustainable soil management [17]. For example, a more adjusted control of the pesticide residues in the EU soils is something that the legislation should address for better monitoring [18]. The noted increasing scarcity over the last decades also raises the need for monitoring of soil salinity levels in the irrigation schemes of southern Portugal [19]. The scientific community can make relevant contributions that can be considered as a basis to design policies that promote proper land management, particularly in terms of risk assessment [20].

Despite the importance of soil quality for sustainable development and the importance of the various policy instruments for preserving soil characteristics, the literature shows that there is room to further explore scientific documents on soil legislation and policies through qualitative and quantitative analyses. In fact, the emphasis by the various stakeholders has been on the air and water dimensions and the soil domains have been neglected [17] and there is no effective system of soil regulation in the EU [6]. This justifies the need for more scientific contributions on these subjects. On the other hand, the approaches considered in this research and the topics addressed emphasise the novelty of this study.

Considering these perspectives, the present study aims to assess soil legislation and policies worldwide, focusing deeper on the Portuguese and EU cases. The objectives are to bring more insights to support the policymakers in Portugal and the EU institutions. More specifically, the objective is to highlight the main trends in the scientific literature on soil policies and legislation, analyse new proposals from the EU in these areas and present proposals to improve the legislation and policy instruments under discussion and those that may be designed.

## 2 Material and methods

A bibliometric analysis was carried out considering documents obtained from the Scopus [21] database and following the procedures proposed by the VOSviewer (software tool for constructing and visualizing bibliometric networks) [[22], [23], [24], [25]] software and the developments of Martinho [26]. On a search performed on April 23, 2024 for the topics “soil legislation*” or "soil polic*", 133 documents were identified. The VOSviewer software was considered to obtain bibliometric networks from text and bibliographic data. Text data were used to obtain co-occurrence links (based on the number of documents in which they appear together) and terms with the most occurrences. Bibliographic data were taken into account to obtain bibliographic coupling links (based on the number of references they share), where items can be authors, countries, documents and sources. With bibliographic data and co-occurrence links were also obtained for the authors' keywords as items. To build these networks, VOSviewer follows a complex methodology described in the manual [24].

After this bibliometric assessment, a systematic review was considered to highlight insights from the literature related to the topics addressed, following the PRISMA (Preferred Reporting Items for Systematic reviews and Meta-Analyses) statement [27]. In practice, 133 documents obtained from the Scopus database were considered. Of these, only 20 studies were analysed in more detail. To select these 20 documents, an approach based on bibliometric analysis was taken into account, selecting the top 20 studies with the highest total link strength (greatest relatedness). The perspective is if the documents have highest relatedness and share the same references, then they are relevant to the topics under analysis.

The EU soil legislation was also surveyed through the ATLAS.ti software [28]. For the analysis with the ATLAS.ti software (data analysis software), qualitative data from the EU Soil Strategy for 2030 and Directive on Soil Monitoring and Resilience proposal were taken into account, which were analysed using quantitative approaches.

## 3 Results

This section is organised into three sub-sections for the bibliometric analysis, systematic review and quantitative evaluation with qualitative data of the EU Soil Strategy for 2030 and Directive on Soil Monitoring and Resilience proposal.

### 3.1 Bibliometric analysis

In this sub-section, tables and figures exhibit the following metrics with the meanings given in the VOSviewer manual [24]: total link strength (total strength of the links between one item and the others); occurrences (total occurrences of a term in all documents for full counting and number of documents in which a keyword appears); average publication year (average publication year of the documents in which a keyword or a term appears or the average publication year of the documents produced by a source, an author, an organisation, or a country); average citations (average number of citations obtained by the documents in which a keyword or a term appears or the average number of citations obtained by the documents produced by a source, an author, an organisation, or a country); average normalised citations (the normalisation deals with the influence of the time in the number of citations); documents (number of documents produced by a source, an author, an organisation, or a country); citations (number of citations obtained by a document); normalised citations (normalised number of citations obtained by a document); and publication year (publication year of a document). On the other hand, the dimensions of the circles and labels are proportional to the number of occurrences (for the terms and author keywords), documents (for the authors, countries and organisations) and citations (for the documents). As well the distance between the circles and labels in the figures is associated with the relatedness (number of documents in which the items appear together for co-occurrence links and the number of references the items share for bibliographic coupling links).

**Fig. 1:**
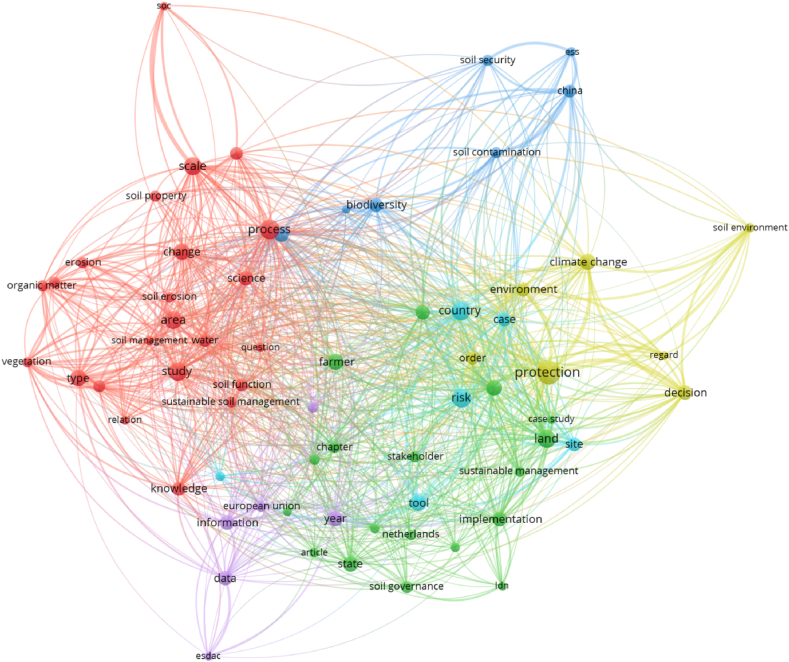
Terms as items for co-occurrence links and text data, considering full counting and 10 as the minimum number of occurrences of a term.

Considering text data and co-occurrence links, Fig. 1 highlights the terms with the highest occurrences for the topics analysed. The terms with the biggest number of occurrences are, for example, the following: protection; process; study; country; risk; area; scale; land; tool; change; type; climate change; and farmer. These terms reveal the importance given by the literature to dimensions associated with soil protection, risks, methodologies and the agricultural sector. Table 1 presents the top terms with the highest total link strength and, in general, there are some similarities with the previous analysis for the number of occurrences.

**Table 1:** Top 20 terms with the highest total link strength, for co-occurrence links and text data, considering full counting and 10 as the minimum number of occurrences of a term.

Terms	Total link strength	Occurrences	Average Publication Year	Average Citations	Average Normalised Citations
protection	1108	76	2016	13	0.554
process	747	49	2016	40	1.312
country	731	46	2016	85	1.665
scale	698	43	2016	65	1.886
risk	621	45	2014	93	1.816
study	606	47	2018	21	1.259
decision	590	29	2013	17	0.474
change	575	34	2015	17	0.815
climate change	557	33	2020	20	0.701
land	557	41	2014	16	0.565
environment	548	25	2014	27	1.002
type	489	34	2013	24	0.857
area	474	44	2014	20	0.832
tool	454	35	2012	58	1.475
concept	432	31	2013	21	0.754
science	424	29	2015	52	1.302
china	413	23	2019	7	0.484
vegetation	411	15	2005	12	1.854
case	402	24	2016	61	1.402
capacity	372	31	2016	22	0.795

**Fig. 2:**
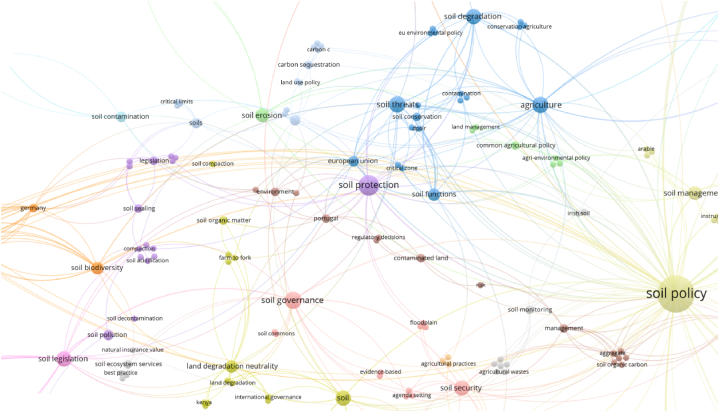
Author keywords as items for co-occurrence links and bibliographic data, considering full counting and 1 as the minimum number of occurrences of a keyword.

The author keywords with the greatest number of occurrences are the following (Fig. 2): soil policy; soil protection; soil health; soil governance; soil threats; agriculture; soil legislation; soil degradation; soil security; soil erosion; soil management; soil biodiversity; and soil functions. There is a visible concern with the soil management to mitigate its degradation, namely in the agricultural practices. The findings in Table 2 for the top author keywords with the greatest total link strength confirm the results obtained for the number of occurrences.

**Table 2:** Top 20 author keywords with the highest total link strength, for co-occurrence links and bibliographic data, considering full counting and 1 as the minimum number of occurrences of a keyword.

Author Keywords	Total link strength	Occurrences	Average Publication Year	Average Citations	Average Normalised Citations
soil policy	189	36	2017	46	1.235
soil protection	51	11	2018	11	0.303
soil threats	49	7	2020	20	0.899
soil health	47	9	2023	4	1.525
soil governance	44	8	2021	8	0.836
soil legislation	43	6	2020	13	0.740
soil biodiversity	37	4	2020	24	1.240
agriculture	32	7	2018	23	0.625
soil degradation	31	6	2016	20	0.712
soil erosion	30	5	2016	29	1.033
soil	29	6	2021	7	0.554
soil security	28	6	2020	26	0.741
soil management	25	5	2018	28	1.062
soil functions	24	4	2016	36	1.252
sustainable soil management	22	4	2023	6	0.361
germany	20	2	2023	5	0.594
soil quality	19	4	2011	45	0.680
land degradation neutrality	18	4	2021	13	1.251
landscape	18	3	2018	43	1.441
soil pollution	18	3	2021	2	0.510

**Fig. 3:**
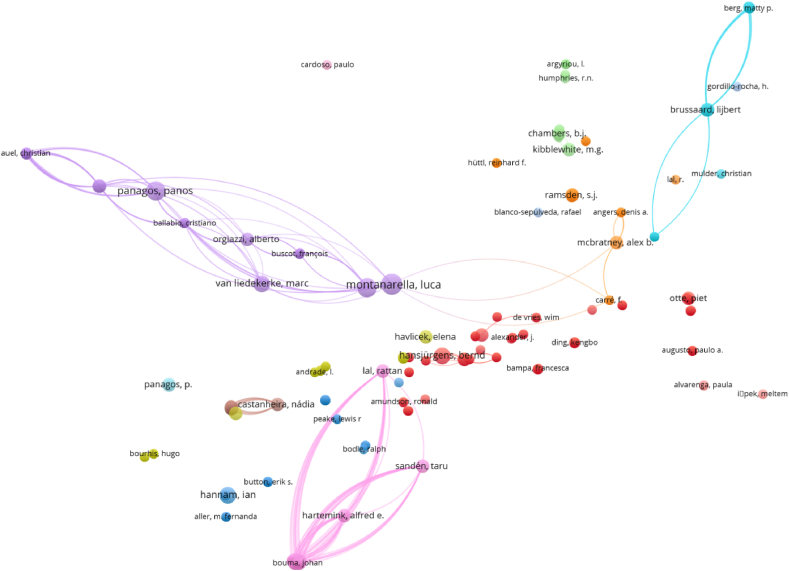
Authors as items for bibliographic coupling links and bibliographic data, considering full counting and 1 as the minimum number of documents of an author.

The most productive authors are Luca Montanarella, Arwyn Jones, Panos Panagos, Marc Van Liedekerke, and Bernd Hansjürgens (Fig. 3). On the other hand, the top authors with the highest total link strength are Taru Sandén, Rattan Lal, Alfred E. Hartemink, Johan Bouma, and Eric Brevik (Table 3). This means that the most productive authors are not those with the greatest relatedness.

**Table 3:** Top 20 authors with the highest total link strength, for bibliographic coupling links and bibliographic data, considering full counting and 1 as the minimum number of documents of an author.

Authors	Total link strength	Documents	Average Publication Year	Average Citations	Average Normalised Citations
Sandén, Taru	5020	2	2019	93	2.965
Lal, Rattan	4447	2	2017	75	1.796
Hartemink, Alfred E.	4194	2	2023	73	1.768
Bouma, Johan	4159	1	2021	145	3.537
Brevik, Eric	4159	1	2021	145	3.537
Dawson, Lorna	4159	1	2021	145	3.537
Field, Damien J.	4159	1	2021	145	3.537
Glaser, Bruno	4159	1	2021	145	3.537
Hatano, Ryusuke	4159	1	2021	145	3.537
Kosaki, Takashi	4159	1	2021	145	3.537
Lascelles, Bruce	4159	1	2021	145	3.537
Monger, Curtis	4159	1	2021	145	3.537
Muggler, Cristine	4159	1	2021	145	3.537
Ndzana, Georges Martial	4159	1	2021	145	3.537
Norra, Stefan	4159	1	2021	145	3.537
Pan, Xicai	4159	1	2021	145	3.537
Paradelo, Remigio	4159	1	2021	145	3.537
Reyes-Sánchez, Laura Bertha	4159	1	2021	145	3.537
Singh, Bal Ram	4159	1	2021	145	3.537
Spiegel, Heide	4159	1	2021	145	3.537

**Fig. 4:**
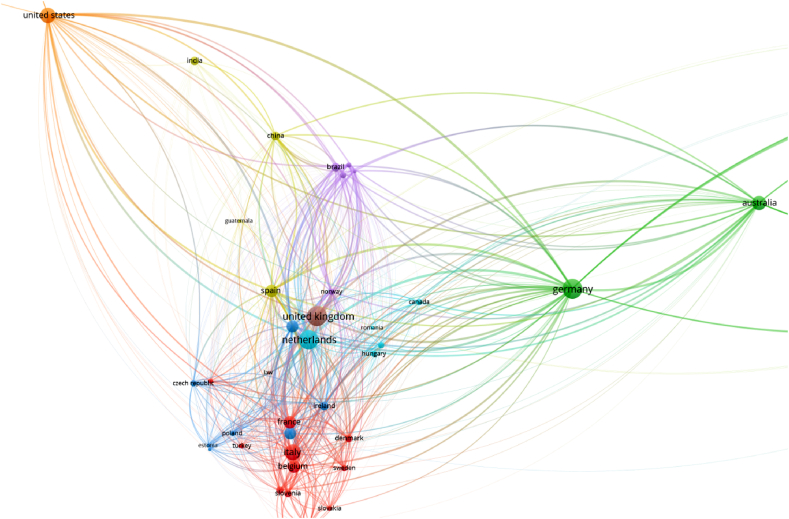
Countries as items for bibliographic coupling links and bibliographic data, considering full counting and 1 as the minimum number of documents of a country.

Germany, the United Kingdom, the Netherlands, Italy, the United States, Australia, Spain and France are the most productive countries (Fig. 4). For the top countries in Table 4, there are some similarities with the findings obtained for the number of documents.

**Table 4:** Top 20 countries with the highest total link strength, for bibliographic coupling links and bibliographic data, considering full counting and 1 as the minimum number of documents of a country.

Countries	Total link strength	Documents	Average Publication Year	Average Citations	Average Normalised Citations
Germany	8122	27	2018	41	1.238
Netherlands	7092	25	2014	47	1.317
Austria	6038	10	2017	58	1.538
Spain	5832	11	2019	46	1.274
United Kingdom	5044	26	2015	41	1.007
France	4716	11	2018	50	1.182
Australia	4661	14	2016	68	1.565
United States	4257	16	2015	67	1.503
Italy	3748	17	2018	46	1.314
Norway	3738	3	2022	51	1.539
Belgium	3620	11	2015	16	0.799
Portugal	2771	10	2019	15	0.801
China	2725	5	2019	44	1.345
Ireland	2536	5	2021	33	1.196
Mexico	2476	3	2017	126	2.435
Brazil	2435	5	2018	41	1.250
Cameroon	2370	1	2021	145	3.537
Japan	2370	2	2021	73	1.768
Slovenia	1932	4	2023	5	0.422
Denmark	1875	4	2017	47	1.745

**Fig. 5:**
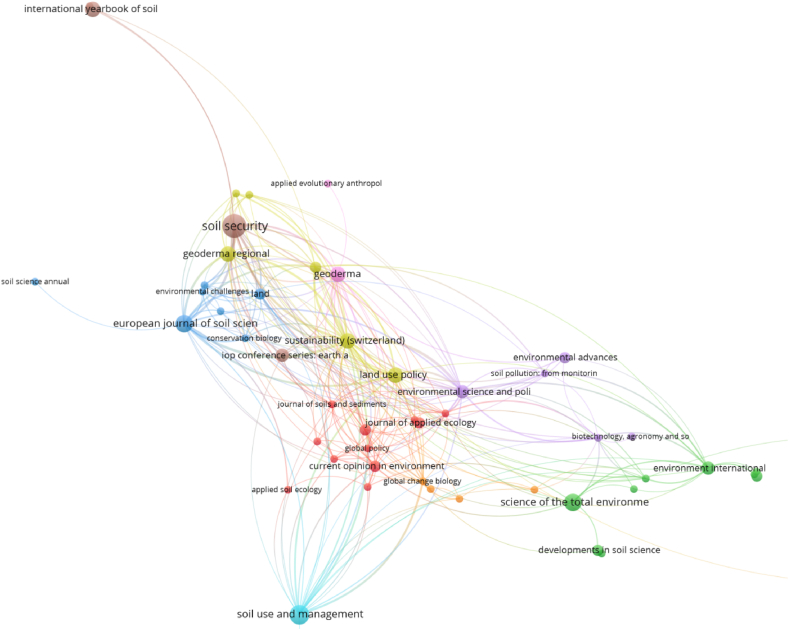
Sources as items for bibliographic coupling links and bibliographic data, considering full counting and 1 as the minimum number of documents of a source.

Soil Security, Soil Use and Management, European Journal of Soil Science; Science of the Total Environment, Sustainability, Geoderma and Land Use Policy are between the sources with the highest number of documents (Fig. 5) and total link strength (Table 5).

**Table 5:** Top 20 sources with the highest total link strength, for bibliographic coupling links and bibliographic data, considering full counting and 1 as the minimum number of documents of a source.

Sources	Total link strength	Documents	Average Publication Year	Average Citations	Average Normalised Citations
Sustainability (Switzerland)	144	4	2017	40	1.702
Soil Security	98	9	2023	5	0.568
European Journal of Soil Science	94	5	2023	8	1.052
Environmental Science and Policy	74	3	2015	10	1.053
Geoderma	67	4	2017	41	1.381
Land Use Policy	63	4	2014	135	1.771
Geoderma Regional	61	4	2022	39	1.337
Journal of Cleaner Production	61	2	2021	33	2.034
Soil Use and Management	61	6	2011	43	0.857
Global Change Biology	39	1	2015	125	3.222
Land	39	2	2021	10	0.481
Environment International	31	3	2012	120	2.105
Environmental Science and Technology	29	1	2011	24	1.600
Journal of Soils and Sediments	28	1	2018	22	1.069
Soil	25	2	2019	79	1.974
Current Opinion in Environmental Sustainability	24	2	2012	39	0.430
Conservation Biology	23	1	2022	10	1.188
Global Policy	23	1	2013	224	3.212
European Journal of Soil Biology	22	1	2012	39	0.435
Romanian Agricultural Research	22	1	2019	0	0.000

The top 20 documents, with the highest total link strength, for bibliographic coupling links and bibliographic data, are presented in Table 6. These documents will be considered for systematic review in the next sub-section.

**Table 6:** Top 20 documents with the highest total link strength, for bibliographic coupling links and bibliographic data, considering full counting and 0 as the minimum number of citations of a document.

Documents	DOI	Total link strength	Citations	Normalised Citations	Publication Year
Vrebos (2017) [13]	https://doi.org/10.3390/su9030407	72	40	2.393	2017
El Hourani (2023) [29]	https://doi.org/10.1016/j.soisec.2023.100100	64	0	0.000	2023
Castelo-Grande (2018) [17]	https://doi.org/10.1016/j.envsci.2017.10.010	57	15	0.729	2018
Glæsner (2014) [6]	https://doi.org/10.3390/su6129538	54	79	2.469	2014
Juerges (2018) [30]	https://doi.org/10.1016/j.jclepro.2016.10.143	52	64	3.111	2018
Lal (2021) [31]	https://doi.org/10.1016/j.geodrs.2021.e00398	44	145	3.537	2021
O'rourke (2015) [32]	https://doi.org/10.1111/gcb.12959	39	125	3.222	2015
Dietze (2019) [33]	https://doi.org/10.1016/j.landusepol.2018.11.003	34	14	0.269	2019
Panagos (2022) [11]	https://doi.org/10.1111/ejss.13315	33	35	4.158	2022
Paz (2024) [34]	https://doi.org/10.1111/ejss.13468	32	0	0.000	2024
Gonzalez Lago (2019) [35]	https://doi.org/10.1016/j.geoderma.2019.04.021	32	8	0.154	2019
Helming (2018) [36]	https://doi.org/10.3390/su10124432	29	20	0.972	2018
Bone (2011) [5]	https://doi.org/10.1021/es101463y	29	24	1.600	2011
Ding (2018) [37]	https://doi.org/10.1007/s11368-016-1547-6	28	22	1.069	2018
Ingram (2022) [38]	https://doi.org/10.3390/land11050599	28	6	0.713	2022
Rodrigues (2009a) [39]	https://doi.org/10.1016/j.envint.2008.08.007	25	64	0.781	2009
Rodrigues (2009b) [20]	https://doi.org/10.1016/j.envint.2008.08.012	25	25	0.305	2009
Weninger (2024) [12]	https://doi.org/10.1111/ejss.13476	23	0	0.000	2024
Koch (2013) [40]	https://doi.org/10.1111/1758-5899.12096	23	224	3.212	2013
Kibblewhite (2016) [41]	https://doi.org/10.1111/sum.12236	23	9	0.225	2016

### 3.2 Systematic review based on bibliometric analysis

Soil is an important resource for sustainable development that requires a specific policy framework to address its challenges, for which solutions need to be found for more effective soil protection [5]. The design of adjusted policies and legislation is crucial to preserve the soil quality, nonetheless, in some circumstances, such as in the Portuguese context, priority was given, over several years, to the air and water conditions [17]. These conditions have promoted a gap between the soil policies in Portugal and other international realities [20]. Additionally, the lack of an adjusted soil regulatory system was also identified for the EU framework [6]. On the other hand, there is a complex interaction between the international, national and regional soil policies [13], and this may hamper an effective policy implementation. The interrelationships among stakeholders and the knowledge dissemination about soil management still need to be improved [12]. In any case, the EU has been creating strategies and regulatory systems to deal with the needs of more healthy soil, including through the creation of a data centre to provide information and knowledge to support the policy design processes [11]. Another strategy frequently used in European Commission projects is stakeholder consultation. An example of this is the series of consultations with soil-related stakeholders, aimed at assessing the current context of knowledge use and the gaps regarding the sustainability of soil management in Europe, carried out by the European Joint Programme for Agricultural Soils (EJP SOIL). The main soil challenges perceived by stakeholders differ across geographic regions of Europe. In Southern Europe, where Portugal is located, the priority challenge is the need to improve soil water storage capacity, while in the rest of Europe, the conservation of soil organic matter and peat soils is the priority [42]. From these EU consultations involving several hundred soil-related stakeholders, including a high representation from the scientific community, soil salinisation was considered a concern mainly in the southern regions of Europe, where the threat is more evident.

The legislation and policy instruments are crucial to foster practices compatible with sustainable development, including in agriculture. In these contexts, the concept of ecosystem services has received more attention from policymakers and the scientific community [33]. Food, water, energy and biodiversity are some of the domains related to the ecosystem services supplied by soils [37]. The ecosystem services are particularly relevant in some specific frameworks, such as the floodplains, however not always adequately preserved [29]. The issues related to soil security are multidisciplinary and this calls for transdisciplinary approaches, an adjusted policy design and an effective policy implementation [35], namely for a more sustainable soil management [36]. Soil security, in certain cases, is considered a central concept in the soil policy design process [40] with soil organic carbon being referred to as an important indicator [32]. Agricultural extension services play an important role in more sustainable use of the soils in Europe, but adjustments are still needed to improve the capacity of these services to support an effective transition to sustainable soil use [38] and a balanced bioeconomy [30]. The scientific community may provide relevant insights for healthier soil use, specifically through quantitative models to assess the policy measures impacts [41]. Sustainable soil practices may contribute significantly to meeting the Sustainable Development Goals of the United Nations [31], but more knowledge is still needed [34]. The implementation of soil policies in the EU is challenging because of the specificities of each country and region [39].

The main insights from some of these documents are summarised and presented in Table 7.

**Table 7:** Main insights from the documents with the highest total link strength.

Documents	DOI	Main insights
Vrebos (2017) [13]	https://doi.org/10.3390/su9030407	Difficult relations among policies at different spatial levels
El Hourani (2023) [29]	https://doi.org/10.1016/j.soisec.2023.100100	Ecosystem services are not properly preserved in some circumstances
Castelo-Grande (2018) [17]	https://doi.org/10.1016/j.envsci.2017.10.010	Priority given by the regulatory system to air and water contexts
Glæsner (2014) [6]	https://doi.org/10.3390/su6129538	Lack of an adjusted soil regulatory system for the European Union framework
Juerges (2018) [30]	https://doi.org/10.1016/j.jclepro.2016.10.143	Soil management and bioeconomy
Lal (2021) [31]	https://doi.org/10.1016/j.geodrs.2021.e00398	Soil practices and Sustainable Development Goals
O'rourke (2015) [32]	https://doi.org/10.1111/gcb.12959	Soil organic carbon is an important indicator for soil assessments
Dietze (2019) [33]	https://doi.org/10.1016/j.landusepol.2018.11.003	Ecosystem services importance
Panagos (2022) [11]	https://doi.org/10.1111/ejss.13315	Soil data centre to support policies design
Paz (2024) [34]	https://doi.org/10.1111/ejss.13468	Knowledge relevance for healthy soil practices
Gonzalez Lago (2019) [35]	https://doi.org/10.1016/j.geoderma.2019.04.021	Multidisciplinary approaches in the soil policy design
Helming (2018) [36]	https://doi.org/10.3390/su10124432	Sustainable soil management analysis
Bone (2011) [5]	https://doi.org/10.1021/es101463y	Soil protection and soil policies
Ding (2018) [37]	https://doi.org/10.1007/s11368-016-1547-6	Ecosystem services provided by soils
Ingram (2022) [38]	https://doi.org/10.3390/land11050599	Extension services and soil sustainability
Rodrigues (2009a) [39]	https://doi.org/10.1016/j.envint.2008.08.007	Soil policies and local specificities
Rodrigues (2009b) [20]	https://doi.org/10.1016/j.envint.2008.08.012	Gaps in the soil policies in Portugal and other contexts
Weninger (2024) [12]	https://doi.org/10.1111/ejss.13476	Interaction between stakeholders and soil knowledge spread
Koch (2013) [40]	https://doi.org/10.1111/1758-5899.12096	Soil security and regulatory system
Kibblewhite (2016) [41]	https://doi.org/10.1111/sum.12236	Importance of quantitative models for the policy impacts analysis

### 3.3 European Union Soil Strategy for 2030 and Directive on Soil Monitoring and Resilience proposal

**Fig. 6:**
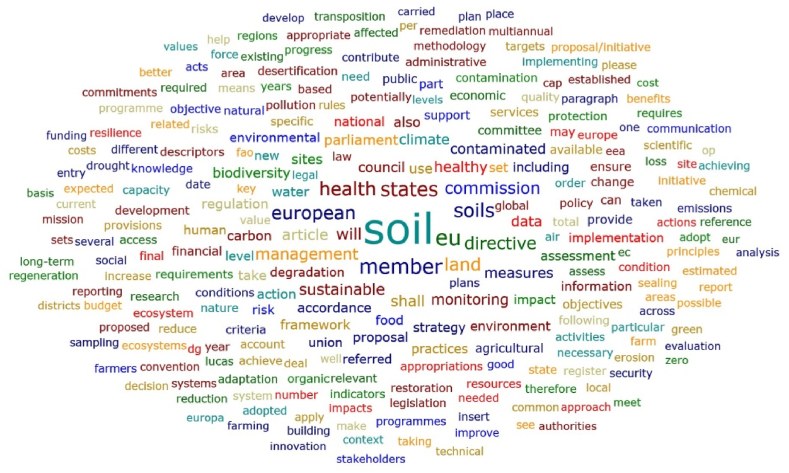
Word cloud considering the European Union Soil Strategy for 2030 document and Soil Monitoring Law proposal.

The words with the highest total absolute counts in the Soil Strategy for 2030 [43] and Soil Monitoring Law (proposal) [44] documents are, for example, the following (Fig. 6): soil; states; health; land; management; sustainable; monitoring; data; contaminated; sites; biodiversity; water; carbon; food; strategy; environment; level; degradation; risk; information; and agricultural. These findings seem to highlight a concern of the Soil Strategy for 2030 document and Soil Monitoring Law proposal with the soil health, land sustainable development and adjusted soil management to mitigate risks and preserve biodiversity. The importance of the information, knowledge and data was also referred. However, when the overall results (exported from the ATLAS.ti software) are analysed, some important words related to relevant dimensions appear less frequently, such as the following: innovation; smart; artificial; CSA (Climate-Smart Agriculture); digital; model; modelling; solution; salinisation. Other relevant words mentioned in the literature do not appear in these documents (for example, multidisciplinary and transdisciplinary). It seems that important threats to soil health, such as salinisation, received less attention in the Soil Strategy for 2030 and in the Soil Monitoring Law proposal, as well as, important current approaches to improve the soil practices related to the digital transition and the concept of Climate-Smart Agriculture (referred as a way to improve the efficiency of the agricultural practices). On the other hand, the focus seems to be more centred in the problems and less in the solutions.

**Fig. 7:**
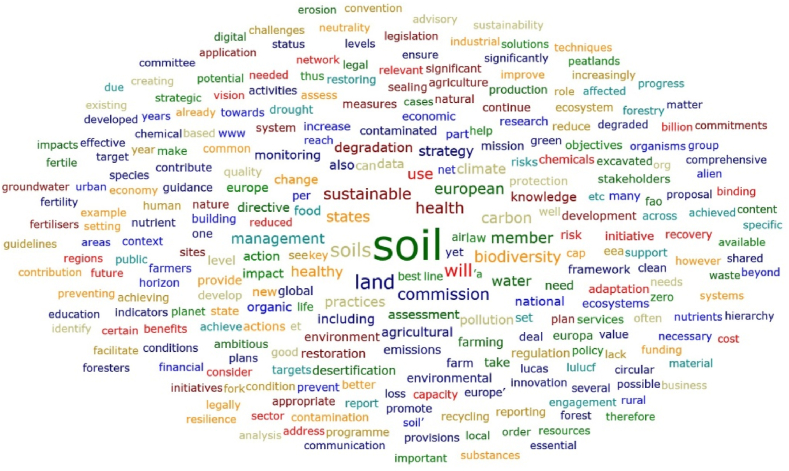
Word cloud considering the European Union Soil Strategy for 2030 document.

**Fig. 8:**
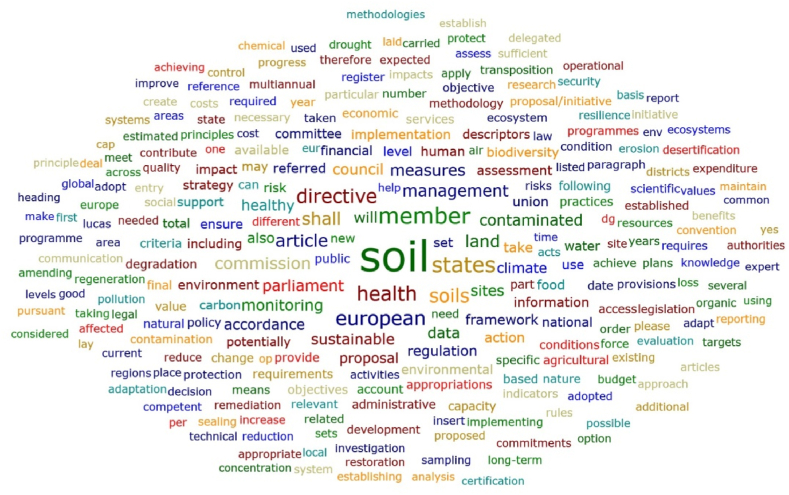
Word cloud considering the Soil Monitoring Law proposal.

Fig. 6, Fig. 7, Fig. 8 and Table 8 reveal that, in general, the highest ranked words in the Soil Strategy for 2030 do not match those in the Soil Monitoring Law proposal. This is confirmed by the Spearman's rank correlation coefficients [[45], [46], [47], [48]] in Table 9 for the overall information exported by the ATLAS.ti software. For example, in the EU Soil Strategy for 2030, the word biodiversity appears more times and with a higher relative count, as does the word carbon. In the Soil Monitoring Law proposal (including annexes) the words shall, management, risk, water and contaminated, for instance, occur more times. These findings deserve further discussion in future research. Albeit, a great match between the words of these documents is not required, and maybe not even expected, a stronger correlation could be a sign of higher relationships between the strategy and the law. The dimensions of the documents are significantly different and this also contributes to the differences identified. These differences in visibility of some concepts can be ascribed to the fact that this Directive has a more practical approach, aiming to implement measures for monitoring and assessing soil health. Additionally, to facilitate the transition towards healthy soils by 2030, the Mission “A Soil Deal for Europe” [49] will finance a series of research and intervention actions.

**Table 8:** Top 50 words with the highest total absolute counts, considering the European Union Soil Strategy for 2030 document and Soil Monitoring Law proposal.

Word	EU Soil Strategy for 2030		Soil Monitoring Law		Soil Monitoring Law (Annexes)		Total	
	Absolute Counts	Relative Counts	Absolute Counts	Relative Counts	Absolute Counts	Relative Counts	Absolute Counts	Relative Counts
soil	314	2.73 %	528	1.92 %	116	3.15 %	958	3.55 %
member	48	0.42 %	224	0.82 %	16	0.43 %	288	1.07 %
states	43	0.37 %	211	0.77 %	10	0.27 %	264	0.98 %
soils	85	0.74 %	153	0.56 %	13	0.35 %	251	0.93 %
health	55	0.48 %	174	0.63 %	13	0.35 %	242	0.90 %
land	86	0.75 %	93	0.34 %	34	0.92 %	213	0.79 %
directive	21	0.18 %	165	0.60 %	11	0.30 %	197	0.73 %
commission	54	0.47 %	123	0.45 %	2	0.05 %	179	0.66 %
management	40	0.35 %	105	0.38 %	15	0.41 %	160	0.59 %
sustainable	53	0.46 %	92	0.33 %	2	0.05 %	147	0.54 %
article	4	0.03 %	134	0.49 %	6	0.16 %	144	0.53 %
shall	0	0.00 %	118	0.43 %	24	0.65 %	142	0.53 %
healthy	40	0.35 %	81	0.30 %	6	0.16 %	127	0.47 %
monitoring	21	0.18 %	93	0.34 %	7	0.19 %	121	0.45 %
measures	11	0.10 %	98	0.36 %	11	0.30 %	120	0.44 %
data	21	0.18 %	92	0.33 %	2	0.05 %	115	0.43 %
contaminated	12	0.10 %	87	0.32 %	14	0.38 %	113	0.42 %
climate	34	0.30 %	69	0.25 %	6	0.16 %	109	0.40 %
parliament	4	0.03 %	97	0.35 %	4	0.11 %	105	0.39 %
sites	11	0.10 %	76	0.28 %	14	0.38 %	101	0.37 %
council	4	0.03 %	90	0.33 %	5	0.14 %	99	0.37 %
biodiversity	49	0.43 %	44	0.16 %	5	0.14 %	98	0.36 %
regulation	16	0.14 %	63	0.23 %	13	0.35 %	92	0.34 %
water	40	0.35 %	33	0.12 %	18	0.49 %	91	0.34 %
carbon	47	0.41 %	36	0.13 %	5	0.14 %	88	0.33 %
accordance	2	0.02 %	72	0.26 %	13	0.35 %	87	0.32 %
action	20	0.17 %	58	0.21 %	7	0.19 %	85	0.32 %
framework	15	0.13 %	69	0.25 %	1	0.03 %	85	0.32 %
proposal	7	0.06 %	76	0.28 %	1	0.03 %	84	0.31 %
food	23	0.20 %	56	0.20 %	0	0.00 %	79	0.29 %
strategy	32	0.28 %	43	0.16 %	1	0.03 %	76	0.28 %
assessment	20	0.17 %	49	0.18 %	6	0.16 %	75	0.28 %
national	23	0.20 %	42	0.15 %	9	0.24 %	74	0.27 %
practices	35	0.30 %	36	0.13 %	3	0.08 %	74	0.27 %
environment	18	0.16 %	51	0.19 %	4	0.11 %	73	0.27 %
including	27	0.23 %	39	0.14 %	5	0.14 %	71	0.26 %
environmental	17	0.15 %	49	0.18 %	4	0.11 %	70	0.26 %
level	19	0.16 %	42	0.15 %	8	0.22 %	69	0.26 %
degradation	30	0.26 %	33	0.12 %	5	0.14 %	68	0.25 %
risk	16	0.14 %	33	0.12 %	19	0.52 %	68	0.25 %
human	10	0.09 %	51	0.19 %	4	0.11 %	65	0.24 %
impact	20	0.17 %	44	0.16 %	0	0.00 %	64	0.24 %
information	3	0.03 %	51	0.19 %	10	0.27 %	64	0.24 %
new	20	0.17 %	39	0.14 %	1	0.03 %	60	0.22 %
implementation	5	0.04 %	53	0.19 %	0	0.00 %	58	0.22 %
provide	18	0.16 %	37	0.14 %	2	0.05 %	57	0.21 %
agricultural	25	0.22 %	29	0.11 %	2	0.05 %	56	0.21 %
change	21	0.18 %	30	0.11 %	4	0.11 %	55	0.20 %
financial	7	0.06 %	48	0.18 %	0	0.00 %	55	0.20 %

**Table 9:** Spearman's rank correlation coefficients between the European Union Soil Strategy for 2030 and the Soil Monitoring Law.

	EU Soil Strategy for 2030	Soil Monitoring Law	Soil Monitoring Law (Annexes)
EU Soil Strategy for 2030	1.000		
Soil Monitoring Law	0.142^a^	1.000	
	(0.000)		
Soil Monitoring Law (Annexes)	0.060^a^	0.084^a^	1.000
	(0.000)	(0.000)	

Note.

a , statistically significant at 1 %.

## 4 Discussion

The bibliometric analysis highlights the concerns of the diverse stakeholders regarding the threats of farming practices to healthy soil and the importance of adjusted land management to avoid soil degradation problems. This is also important to preserve the biodiversity and the soil functions. There is, in fact, an interconnection between agricultural practices and soil quality that is very important for them to remain sustainable. Soil policies and legislation play an important role here. Soil policy motivations at the European level have been increasing, as showed by the EU Soil Strategy 2030, the European Green Deal, the EU Soil Observatory and the recent Soil Monitoring and Resilience Directive proposal. Several regulatory systems have addressed soil and its threats, although no EU-wide legislation explicitly focusing on soil. With this objective, the Soil Monitoring Law proposal was created aiming to implement measures for monitoring and analysing soil health, and to stimulate sustainable soil management while dealing with contaminated sites [[50], [51], [52]]. In this recent Directive, a list of soil descriptors and soil health criteria is proposed to be measured and monitored by Member States, allowing some flexibility to adapt to national or local conditions. Such flexibility might be well received by Member States, since it opens the possibility of choosing indicators and consider thresholds adapted to the national pedoclimatic conditions. On the other hand, the most productive authors are not those with the highest total link strength for bibliographic coupling links. This means that the documents from the most productive authors may be not those with a higher number of references shared. This shows that the choice of metrics to use in bibliometric analysis must be adjusted to the intended objectives. The most productive countries are, for example, the following: Germany; the United Kingdom; the Netherlands; Italy; the United States; Australia; Spain; and France. Finally, the sources with the highest number of documents seem to be focused on soil, environment, sustainability and land policy.

The systematic review revealed the importance of making the soil regulatory systems compatible at international, national and regional levels. Additionally, it is important to recognise the importance of the ecosystem services. In general, it was highlighted the priority given, over the last decades, to air and water policies and legislation and the need for a more adjusted soil legal framework in the EU context. Soil security is central to the policy design processes and carbon is an important indicator. Knowledge and data may provide valuable insights, along with multidisciplinary approaches, well-prepared extension services, stakeholders interactions and quantitative models for policy impact assessment. There are some soil databases at the European scale, although more extensive data is available in individual European countries, allowing a more exhaustive soil analysis. In order to evaluate existing agricultural soil data sources and combining data at the European scale, the European Joint Programme on Agricultural Soil Management (EJP SOIL) created by the European Commission, conducted a survey in 2020 across 24 European member-states. This survey showed that most of the main pedological and chemical indicators are included in more than 70 % of the member-state soil databases, nonetheless water characteristics, contamination through organic pollutants, and biological indicators are the least times referred [53]. These differences will have an impact on the design of EU soil policy instruments and definition of parameters. Differences in the approaches used to assess soils are another concern, showing the need for harmonisation of methodologies and more collaboration between member-states and with the EU. The LUCAS survey (Land Use and Coverage Area frame Survey) [54] already provides harmonised information on topsoil in the context of the EU, but improvements are still needed. For example, monitoring soils at depths greater than 20 cm is critical for assessment of soil threats such as soil salinisation. Additionally, it is important to address the gaps in knowledge about soil in EU countries [55]. In any case, it seems that the biggest concern should be finding ways to bring the various stakeholders into agreement, at national and EU level, to define an effective and consensual regulatory system for soils.

The EU Soil Strategy for 2030 and Soil Monitoring Law proposal assessment, namely through the word cloud and word list obtained with the ATLAS.ti software, displayed also the need for sustainable soil management and the importance of knowledge in these processes. On the other hand, some relevant issues, such as salinisation, innovation, smart methodologies, modelling, new solutions and transdisciplinary approaches, seem to be ignored, or at least received less attention. The threats and challenges to soil are particular to the different European regions, so the transposition of European soil policies into the national regulatory system must be carried out taking into account Portugal's specific pedoclimatic and socioeconomic conditions. In Portugal, there is no nationwide soil monitoring system, like in other EU countries [20]. There is a need to better understand the soil knowledge needs in Portugal identified by stakeholders, as well as the definition of indicators and thresholds. The compatibility of the Portuguese INFOSOLO [56,57] database with the LUCAS survey can provide relevant insights for defining these parameters.

## 5 Conclusions

In terms of practical implication, it's worth mentioning that the soil regulatory system has gained more notoriety in the last years, but there is still a long way to go. The different related stakeholders are focused on soil health, protection and security. There is also a concern with the land sustainable development and management, particularly in mitigating risks, preserving biodiversity and maintaining ecosystem services. However, there is a field to be explored, specifically addressing dimensions associated with the multidisciplinary and smart approaches, soil salinisation, innovation and quantitative models for policy impacts analysis.

For policy recommendation and to improve the tools and measures of the soil policy of Portugal and the EU, it could be important to design instruments that foster the interaction between stakeholders, transdisciplinary research and the adoption of artificial intelligence methodologies in soil management and monitoring. The problems associated with soil salinisation are real and need to be addressed effectively. The EU regulatory framework for soil must be clear in describing the problem, identifying the solutions and how the various instruments and measures are to be applied.

To enhance assessments of soil governance within the EU framework, future research should explore the potential of machine learning techniques leveraging existing datasets. This could involve developing predictive models to identify areas at risk of soil degradation and analysing policy interactions for a more holistic approach. It will also be important to understand what the different stakeholders have to say on the subject in order to find clues for improving the soil regulatory system. These are limitations that could be explored in future studies.

## Data availability statement

Data will be made available on request.

## CRediT authorship contribution statement

**Vítor João Pereira Domingues Martinho:** Writing – review & editing, Writing – original draft, Visualization, Validation, Supervision, Software, Resources, Project administration, Methodology, Investigation, Funding acquisition, Formal analysis, Data curation, Conceptualization. **António José Dinis Ferreira:** Writing – review & editing, Visualization, Validation, Investigation, Funding acquisition, Formal analysis, Conceptualization. **Carlos Cunha:** Writing – review & editing, Visualization, Validation, Investigation, Funding acquisition, Formal analysis, Conceptualization. **José Luís da Silva Pereira:** Writing – review & editing, Visualization, Validation, Investigation, Funding acquisition, Formal analysis, Conceptualization. **María del Carmen Sánchez Carreira:** Writing – review & editing, Visualization, Validation, Investigation, Funding acquisition, Formal analysis, Conceptualization. **Nádia Luísa Castanheira:** Writing – review & editing, Visualization, Validation, Investigation, Funding acquisition, Formal analysis, Conceptualization. **Tiago Cunha Brito Ramos:** Writing – review & editing, Visualization, Validation, Investigation, Funding acquisition, Formal analysis, Conceptualization.

## Declaration of competing interest

The authors declare the following financial interests/personal relationships which may be considered as potential competing interests: Vítor Martinho is Associate Editor of the Heliyon Journal. This fact did not affect the peer-review process.
